# Can Potato Plants Be Colonized with the Fungi *Metarhizium* and *Beauveria* under Their Natural Load in Agrosystems?

**DOI:** 10.3390/microorganisms9071373

**Published:** 2021-06-24

**Authors:** Maksim Tyurin, Marsel R. Kabilov, Natalia Smirnova, Oksana G. Tomilova, Olga Yaroslavtseva, Tatyana Alikina, Viktor V. Glupov, Vadim Yu Kryukov

**Affiliations:** 1 Institute of Systematics and Ecology of Animals, Siberian Branch of the Russian Academy of Sciences, 630091 Novosibirsk, Russia; toksina@mail.ru (O.G.T.); yarosl@inbox.ru (O.Y.); skif61@list.ru (V.V.G.); 2 Institute of Chemical Biology and Fundamental Medicine, Siberian Branch of the Russian Academy of Sciences, 630090 Novosibirsk, Russia; kabilov@niboch.nsc.ru (M.R.K.); alikina@niboch.nsc.ru (T.A.); 3 Institute of Soil Science and Agrochemistry, Siberian Branch of the Russian Academy of Sciences, 630090 Novosibirsk, Russia; nat-smirnova@yandex.ru

**Keywords:** endophytes, ascomycetes, ITS, metagenomics, fungal communities, rhizosphere

## Abstract

*Beauveria* and *Metarhizium* fungi are facultative plant endophytes that provide plant growth-stimulating, immunomodulatory, and other beneficial effects. However, little is known about the level of plant colonization by these fungi under natural conditions. We assessed the endophytic colonization of potatoes (*Solanum tuberosum*) with entomopathogenic fungi at their natural load in soils (10^2^–10^4^ colony-forming units per g). Microbiological analyses of soils and plant organs, as well as a metagenomic analysis of potato roots and leaves, were conducted in three locations in Western Siberia, consisting of conventional agrosystems and kitchen gardens. The fungi were isolated at a relatively high frequency from unsterilized roots (up to 53% of *Metarhizium*-positive plants). However, the fungi were sparsely isolated from the internal tissues of roots, stems, and leaves (3%). Among the genus *Metarhizium*, two species, *M. robertsii* and *M. brunneum,* were detected in plants as well as in soils, and the first species was predominant. A metagenomic analysis of internal potato tissues showed a low relative abundance of *Beauveria* and *Metarhizium* (<0.3%), and the communities were represented primarily by phytopathogens. We suggest that colonization of the internal tissues of potatoes occurs sporadically under a natural load of entomopathogenic fungi in soils. The lack of stable colonization of potato plants with *Beauveria* and *Metarhizium* may be due to competition with phytopathogens.

## 1. Introduction

Entomopathogenic fungi from the genera *Beauveria* and *Metarhizium* are natural regulators of insect populations and are environmentally friendly agents used for pest control [1]. The primary reservoirs of these fungi are the soil and plant rhizosphere. Over the last two decades, these fungi have been shown to have a multifunctional lifestyle. In particular, *Beauveria* and *Metarhizium* species are unspecialized rhizosphere colonizers and endophytes, and may exert a number of beneficial effects on plants, such as growth promotion, immunity modulation, and antagonistic action towards phytopathogens and herbivores [2]. Many studies have shown the successful colonization of plants after their inoculation with *Beauveria* and *Metarhizium* fungi or after the introduction of these fungi into the soil, as recently reviewed by Vega [3] and Bamisile and coauthors [4]. However, few studies have focused on quantitative analyses of plant colonization under the natural load of fungi in ecosystems. It seems that natural endophytic colonization of grasses and trees by entomopathogenic fungi occurs infrequently (e.g., [5,6,7]).

The potato *Solanum tuberosum* is one of the most important crops in the world. According to FAOSTAT data for 2016, 375 million tons of potatoes are produced globally per year [8]. Potato fields, like other agrosystems, harbor entomopathogenic fungi from the genera *Beauveria, Metarhizium*, and some *Cordyceps* (*C. farinosa*), as shown in potato pest studies [9,10]. Several studies have shown that treating potatoes with entomopathogenic fungi positively affects plants. For example, Rios-Moreno and coauthors [11] performed laboratory experiments to show that spraying potatoes with conidia from *Metarhizium brunneum* strains led to successful colonization of the plants, and that the level of fungal toxins (destruxin A) in the potato tissues was extremely low. Krell and coauthors [12] showed that treating potatoes with *M. brunneum* mycelia formulated in beads and their subsequent cultivation in sterile sand led to the endophytic colonization of various plant organs at 5–45% and an increase in plant biomass. The authors showed that the change in water use efficiency, and nitrogen and phosphorus content in potatoes under the influence of *M. brunneum* treatment depended on the nutrient condition (with and without fertilizers) [13]. The authors suggested that *M. brunneum* may mitigate nutrient deficits in soil by improving plant productivity. We showed in previous work [14] that treating potato tubers pre-planting with *Beauveria bassiana* and *Metarhizium robertsii* conidia led to a decrease in *Rhizoctonia* disease and stimulation of plant growth under field conditions. Thus, work on the interactions between entomopathogenic fungi and potato plants was conducted either in the laboratory in sterile substrates or under field conditions after introducing the fungi. However, it is not known whether potatoes can be colonized by *Beauveria* and *Metarhizium* under the natural fungal abundance in agrosystems.

The load of entomopathogenic fungi in natural coenoses and agrosystems is usually 10^2^–10^4^ colony-forming units (CFUs) per g soil [15]. The CFU count depends on many biotic and abiotic factors, and the primary factors are the physicochemical properties of the soil (texture, temperature, and humidity regimes), the density of the host insects, and the agricultural practices [16]. In particular, loamy, clay, and silty soils are more suitable for fungal persistence and infectivity towards insects than sandy soils [16]. Using herbicides and fungicides has little or no effect on the fungi in the soil, as shown in various agricultural (non-potato) systems [17,18,19]. However, tillage can dramatically reduce the abundance of *Beauveria* and *Metarhizium* [20] because the conidia are sensitive to UV radiation and high temperatures. The abundance of entomopathogenic fungi in the soil of organic agrosystems is usually greater than that in conventional agrosystems [19,21], which is probably due to the high content of organic matter and the higher density of arthropods. It should be noted that a special study on the abundance of entomopathogenic fungi in the soils of potato plantations has not been conducted.

The aim of this study was to establish the level of potato endophytic colonization by entomopathogenic fungi in plantations under long-term potato cultivation in Western Siberia. Based on the cultivation methods, we revealed the frequency of plant colonization by the fungi and their CFU counts in soils from different locations around the region. In addition, we established the relative abundance of *Metarhizium* and *Beauveria* vs. other fungal genera in potato roots and leaves using MiSeq Illumina sequencing. The species composition of *Metarhizium* fungi isolated from the potato plants and soils was also analyzed by sequencing the elongation factor region (*5’EF-1α*) for 37 isolates.

## 2. Materials and Methods

### 2.1. Locations and Soil Properties

This work was performed during 2019–2020 in Western Siberia at 3 geographical points in the Novosibirsk region (Figure S1):

(1) The neighborhood of Karasuk town (53°729096′ N, 77°650617′ E) in the steppe zone. This is a more arid area than the other locations [22] (Figure S2). The agrosystem consists of kitchen gardens with continuous potato cultivation (more than 10 years). The agricultural practices are domestic, with only 1 mechanized tillage per year before potato planting. The soil is sandy clay that is acidic, with low nitrogen but high phosphorus contents (Tables S1 and S2, Figure S3). There was a high density of the Colorado potato beetle (up to 100 individuals per plant) over 10 years. Irregular applications of insecticides are conducted.
(2) The neighborhood of Novosibirsk city (55°063772′ N, 82°760315′ E) in the forest–steppe zone. This is a conventional agrosystem with crop rotation and intensive farming (more than 40 years). Potatoes are cultivated using Dutch technology [23]. Autumn tillage is also performed. The soil is a sandy clay loam with an alkalescent pH and the lowest nitrogen content compared with the other locations (Tables S1 and S2, Figure S3). Insecticides, herbicides, and fungicides are regularly applied. The density of pest insects is extremely low.
(3) The neighborhood of Toguchin town (55°043662′ N, 84°803181′ E) in the forest–steppe zone. This is the most humid location [22] (Figure S2). The agrosystem is made up of kitchen gardens under continuous potato cultivation (at least 10 years). Potatoes are cultivated in the same way as in the Karasuk location. The soil is silty clay with a neutral pH, with the highest nitrogen and microelement contents compared with the other locations (Tables S1 and S2, Figure S3). The density of Colorado potato beetles is 0–5 larvae per plant.

### 2.2. Sample Collection

All the samples were collected during the potato flowering period (14–15 July 2019 and 19–20 July 2020). Five soil samples were randomly collected from each location at a distance of 15–20 m from each other at a depth of 5–10 cm. Each of the 5 samples was pooled from 3 subsamples collected from a radius of 2–3 m. The soils were placed in plastic bags and delivered to the laboratory for analysis within 24 h. Potato plants were also collected randomly (the distance between plants was 10–12 m). Thirty samples from each location were used. The shoots and roots were packed in plastic bags and delivered to the laboratory for testing within 24 h.

In 2019, an analysis of fungal CFUs in the soils and a microbiological analysis of the endophytic colonization of potato plants were conducted. In 2020, the same analyses were conducted again. In addition, in 2020, a microbiological analysis of the non-sterilized roots and a metagenomic analysis of potato tissues were performed.

### 2.3. Metarhizium and Beauveria CFU Count in Soils

To assess the number of CFUs in bulk soils, 5 g of each sample was suspended in 40 mL of a sterile water–Tween solution (0.1%), vortexed for 10 s, and shaken at 180 rpm for 1 h. A 100 μL aliquot of the soil suspension from each sample was plated in 90 mm Petri dishes with a modified Sabouraud medium (glucose, 40 g/L; peptone, 10 g/L; yeast extract, 1 g/L; agar, 20 g/L) supplemented by cetyltrimethylammonium bromide (0.35 g/L), cycloheximide (0.05 g/L), tetracycline (0.05 g/L), and streptomycin (0.6 g/L) to inhibit the growth of saprophytic fungi and bacteria. The plates were incubated at 25 °C for 14 d, and the *Metarhizium* and *Beauveria* colonies were detected by light microscopy and counted. Representative fungal colonies were selected for molecular biology analysis. A weighed portion of each soil sample was dried at a temperature of 60 °C for 24 h, and the CFU count was adjusted to the dry weight of the soils.

### 2.4. Microbiological Analysis of Plant Colonization

To assess endophytic colonization by the entomopathogenic fungi, we selected the middle part of the root, the lower third of the stem, and the leaf from the middle plant layer. The plant parts were washed with running water and sterilized with 0.5% sodium hypochlorite and 70% ethanol as described by Posada and coworkers [24]. The organs were imprinted [25] on the abovementioned medium and then placed on the surface of the medium in 90 mm Petri dishes. After 14–20 days of incubation, the growth of *Beauveria* and *Metarhizium* was detected visually and by light microscopy. The percentage of fungus-positive plants was then calculated. Samples showing fungal growth on the imprints were excluded from the analysis.

To analyze colonization of the non-sterilized roots, the middle parts of the roots were washed 3 times (1 min at 180 rpm each time) in a water–Tween 20 solution (0.04%) and plated on the abovementioned medium in petri dishes. Incubation and detection of the fungi were performed as described above.

### 2.5. Phylogenetic Analysis of Metarhizium Fungi Isolated from Plants and Soils

Five-day-old fungal cultures grown on Sabouraud agar were collected to isolate their DNA using DNAeasy Plant Mini Kits (Qiagen, Hilden, Germany) according to the manufacturer’s instructions. The amplification of the *5’EF-1α* region was performed using the primers EF1T (5′ TGGGTAAGGARGACAAGAC 3′) and EF2T (5′ GGAAGTACCAGTGATCATGTT 3′) via a technique described previously [26]. Sequencing was performed by the Evrogen company (Moscow, Russia). To construct a phylogenetic tree, GenBank cultures were used, including the type strains of the PARB *Metarhizium* clade [27]. The resulting sequences were compared against GenBank sequences using the built-in BLAST utility and BioEdit software [28]. Phylogenetic reconstructions were performed using the maximum likelihood method and the Tamura–Nei model [29] with 1000 replicates as bootstrap support. This analysis involved 50 nucleotide sequences (37 from this work and 13 from GenBank), resulting in an alignment that was 730 bp long. *Metarhizium lepidotae* and *M. acridum*, not belonging to the PARB clade, constituted the outgroups. Evolutionary analyses were conducted in MEGA X [30].

### 2.6. ITS Metagenomics

Five leaf samples and 5 root samples from each location were used for the analysis. Each sample was pulled from 5 leaves or 5 roots from different plants. The potato plants were washed under running water, and the plant organs were sterilized with 0.5% sodium hypochlorite and 70% ethanol as described by Posada and coauthors [24]. Next, the plants were rinsed with sterile water and frozen at −80 °C until analysis. A conidial mixture of *B. bassiana* (strain Sar-31) and *M. robertsii* (strain P-72) was used as a positive control. The conidia were grown on Sabouraud agar, homogenized in liquid nitrogen, and frozen at −80 °C.

The DNA was isolated using a DNeasy PowerSoil Kit (Qiagen). Bead beating was performed using a TissueLyser II (Qiagen) for 10 min at 30 Hz. The ITS mycobiome was assessed by a nested PCR approach. In the first PCR, the primers ITS1-F_KYO2 [31] and ITS4 [32] were used to suppress the co-amplification of plant-derived ITS regions [33].

The obtained PCR products were used for nested PCR with the primer pair ITS3_KYO2 [33] and ITS4, combined with Illumina adapter sequences [34]. Amplification was performed as described previously [35]. In total, 200 ng of the PCR products from each sample (a mix of 3 technical replicates) was pooled together and purified with a MinElute Gel Extraction Kit (Qiagen). The ITS libraries were sequenced with 2 × 300 bp paired-end reagents on MiSeq (Illumina, CA, USA) in the SB RAS Genomics Core Facility (ICBFM SB RAS, Novosibirsk, Russia).

Raw sequences were analyzed with the UPARSE pipeline [36] using Usearch v11.0.66710.0. The UPARSE pipeline included the merging of paired reads, read quality filtering, length trimming, merging of identical reads (dereplication), discarding singleton reads, removing chimeras, and operational taxonomic unit (OTU) clustering with ≥97% identity using the UPARSE-OTU algorithm. Additionally, the UNCROSS2 algorithm was used in the OTU table to exclude crosstalk errors and assign reads to incorrect samples [37]. The OTU sequences were assigned a taxonomy using the SINTAX [38] with a confidence level of 0.8, and all eukaryotes using ITS UNITE USEARCH/UTAX v.8.2 [39] as a reference.

Rarefaction and extrapolated curves were generated using the “iNEXT” package [40]. All the rarefaction curves had a tendency to approach the saturation plateau (Figure S4), and the average number of reads was 37,366 ± 1255 per sample. However, the final dataset without OTUs belonging to plants (mainly potato plastids and mitochondria) included only 31,571 reads (1052 ± 227 per sample) (see Supplementary Materials File S1).

### 2.7. Statistical Analyses

Data analysis was performed using Statistica 8 (StatSoft Inc., Tulsa, OK, USA) and PAST 3 [41]. The normality of the data distribution was checked using the Shapiro–Wilk W-test. Since the data were not normally distributed, they were analyzed by Kruskal–Wallis ANOVA followed by Dunn’s post-hoc test. Fisher’s exact test was used to assess the difference in the frequency of colonized plants as well as in the frequency of *M. robertsii* and *M. brunneum* in different locations and plant organs.

## 3. Results

### 3.1. CFU Count in Soils

**Figure 1:**
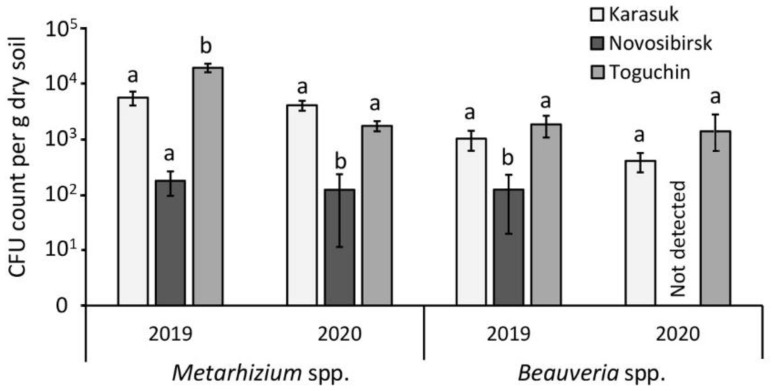
The CFU counts of *Metarhizium* and *Beauveria* in soil samples from plots occupied by domestic potato gardens (Karasuk, Toguchin) and conventional potato fields (Novosibirsk) in 2019 and 2020. Vertical lines indicate the standard errors (SE). Different letters indicate significant differences calculated for *Metarhizium* and *Beauveria* and each year separately (Dunn’s test, *p* < 0.05).

Microbiological analysis of the soils showed that the CFU counts of entomopathogenic fungi in soils from the kitchen gardens of Karasuk (sandy clay soil) and Toguchin (silty clay soil) were close and varied within 2 × 10^3^–2 × 10^4^/g dry soil for *Metarhizium* and 4 × 10^2^–2 × 10^3^/g dry soil for *Beauveria* (Figure 1). A significant difference between these locations was registered only in 2019, when the CFU count of *Metarhizium* in Toguchin was slightly higher than that in Karasuk soil (Dunn’s test, *p* = 0.02). In the conventional agrosystem of Novosibirsk (sandy clay loam soil), the CFU count was one to two magnitudes lower than in the Karasuk and Toguchin soils, and was only 1 × 10^2^/g dry soil for *Metarhizium* and 0–1 × 10^2^/g dry soil for *Beauveria*. In most cases, the lower CFU count in the Novosibirsk location was significant compared with the Karasuk and Toguchin locations. Thus, the CFU count of the fungi was lower in the soils of the conventional agrosystem than in the kitchen gardens, and, in most assays, *Metarhizium* was more abundant than *Beauveria*.

### 3.2. Frequency of Endophytic Colonization of Potato Plants

*Metarhizium* and *Beauveria* fungi were isolated to a very limited extent from surface-sterilized plant roots, stems, and leaves (Table 1). In particular, the average number of *Metarhizium*-positive roots was 3.3%, the number of *Metarhizium*-positive stems was 2.2%, and the number of *Metarhizium*-positive leaves was 0% for both years and for all locations (*n* = 180). Regarding *Beauveria*, this genus was found in 1.7% of roots, 1.7% of stems, and 0.6% of leaves (*n* = 180). No significant differences in the frequency of colonization between or among locations, years of study, and plant organs were found (Fisher’s exact test, *p* > 0.24). However, there was a slight trend towards a decrease in the percentage of colonized plants in the conventional agrosystem of Novosibirsk compared with the kitchen gardens of Karasuk.

### 3.3. Frequency of Isolation from Non-Sterilized Roots

**Figure 2:**
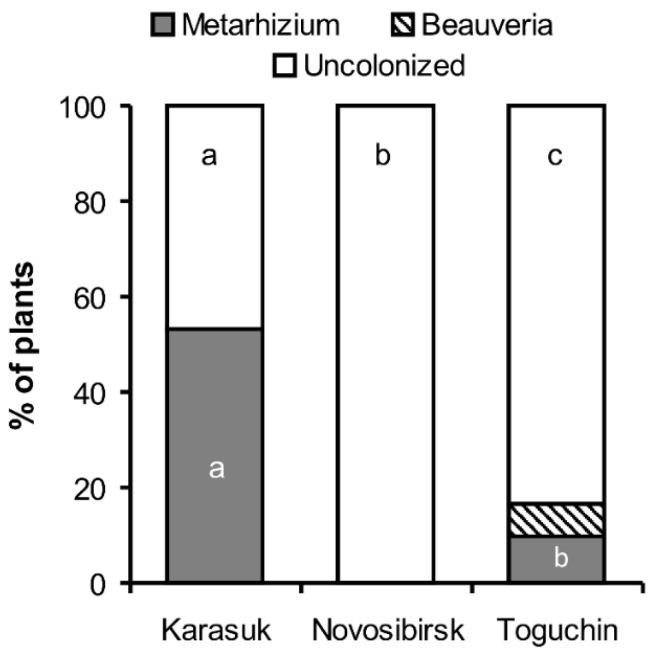
Isolation of *Metarhizium* spp. and *Beauveria* spp. from non-sterilized potato roots from kitchen gardens (Karasuk, Toguchin) and the conventional agrosystem (Novosibirsk) in 2020 (*n* = 30 per point). Different letters indicate significant differences among locations (Fisher’s exact test, *p* < 0.03).

The plating of non-sterilized potato roots on media showed a higher frequency of *Metarhizium-* and *Beauveria*-positive roots than endophytically colonized roots. In particular, the fungi were isolated from 53% of plants from the Karasuk kitchen gardens, 16% of plants from the Toguchin kitchen gardens, and 0% from the Novosibirsk conventional agrosystems (Figure 2). Differences in the proportion of colonized to uncolonized plants were significant between all locations (Fisher’s exact test, *p* < 0.03).

### 3.4. Analysis of Plant Fungal Communities by ITS Metagenomics

**Figure 3:**
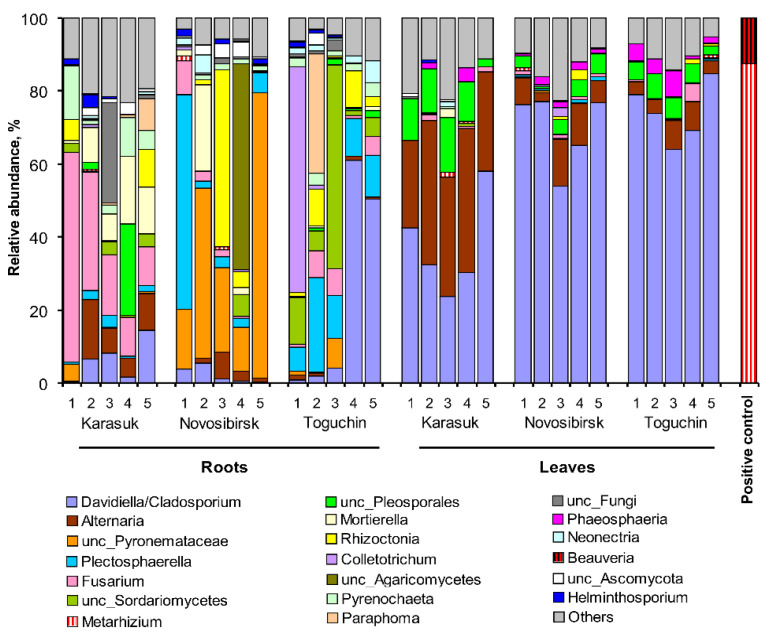
Fungal communities of surface-sterilized potato roots and leaves from kitchen gardens (Karasuk, Toguchin) and a conventional agrosystem (Novosibirsk) in 2020 as estimated by ITS metagenomics sequencing at the genus level. Five samples for each point are presented. The positive control was a mixture of *M. robertsii* and *B. bassiana* conidia. Crosstalk algorithm was not applied.

The metagenomic analysis of surface-sterilized potato roots and leaves revealed 338 OTUs primarily belonging to Ascomycota from the orders Pleosporales, Capnodiales, Hypocreales, and Pezizales (Supplementary Materials File S1). The relative abundance of Basidiomycota was only 6.8%, and the OTUs belonged mostly to Ceratobasidiaceae (Cantharellales). The fungal communities were represented primarily by phytopathogenic and some saprotrophic fungi. The OTUs of *Fusarium, Plectosphaerella, Cladosporium, Rhizoctonia, Mortierella, Colletotrichum*, and unclassified *Pyronemataceae* predominated in the roots (Figure 3), and the OTUs of *Alternaria* and *Cladosporium* were abundant in the leaves. The abovementioned taxa (with the exception of *Pyronemataceae*) included pathogens of potato or other Solonaceae plants (e.g., [42,43]).

Regarding entomopathogens, a very low relative abundance of the studied fungi was registered. The average relative abundance of *Metarhizium* OTUs was 0.2 ± 0.1% in roots and 0.3 ± 0.1% in leaves. There was no significant difference in *Metarhizium* abundance among locations or between roots and leaves (Dunn’s test, *p* > 0.20). *Beauveria* OTUs were not found in the roots, and the average relative abundance in leaves was only 0.008 ± 0.005%. It should be noted that the detection of *Metarhizium* and *Beauveria* DNA in the internal potato tissues could be false positives. In particular, we did not detect *Metarhizium* and *Beauveria* reads in the studied samples when the crosstalk algorithm was applied. The equal *Metarhizium* abundance in the leaves and roots (if the crosstalk algorithm was not applied) may also indicate false positive results. However, in any case, the participation of *Metarhizium* and *Beauveria* in the potato endophyte community was exceptionally low.

The structure of the communities of phytopathogenic fungi varied significantly depending on the location. In particular, the highest abundance of *Fusarium* was observed in roots from Karasuk (Dunn’s test, *p* ˂ 0.005 compared with the other locations). The OTUs of unclassified Pyronemataceae (Ascomycota, Pezizales) predominated in potato roots from the conventional agrosystem of Novosibirsk (*p* ≤ 0.02 compared with the other locations). Interestingly, this OTU had 100% shared identity only with the uncultured fungus found in the rhizosphere of tomatoes in Mexico [44]. There was a strong irregular abundance of taxa between replicates in the roots of the Toguchin location. Nevertheless, the abundance of *Neonectria* and *Plectosphaerella* increased, and the abundance of *Alternaria* and *Mortierella* decreased significantly in potato roots from Toguchin compared with Karasuk (*p* ≤ 0.03), but not in those from the Novosibirsk location. No significant difference was revealed among locations in terms of the abundance of the phytopathogenic fungi *Rhizoctonia, Cladosporium*, and *Colletotrichum*.

In potato leaves, significant differences among the fungal communities from different locations were also registered. In particular, the relative abundance of *Alternaria* increased and that of *Cladosporium* decreased in the leaves from Karasuk compared with the other locations (*p* ˂ 0.03). However, no significant difference was found between the Novosibirsk and Toguchin locations in the ratio of *Alternaria* to *Cladosporium*.

It should be noted that in addition to phytopathogenic and saprotrophic fungi, a small amount (8–16 reads) of nematode pathogenic fungi was detected in the plants. In particular, OTU 474 from potato roots was 100% identical to *Metacordyceps* (=*Pochonia) chlamydosporia* strains (Hypocreales, Clavicipitaceae). OTU 296 from roots and leaves was identical (100%) to *Hirsutella rhossiliensis* (Hypocreales, Ophiocordycipitaceae) cultures from the United States [45].

### 3.5. Identification of Metarhizium Species Isolated from Soils and Plants

**Figure 4:**
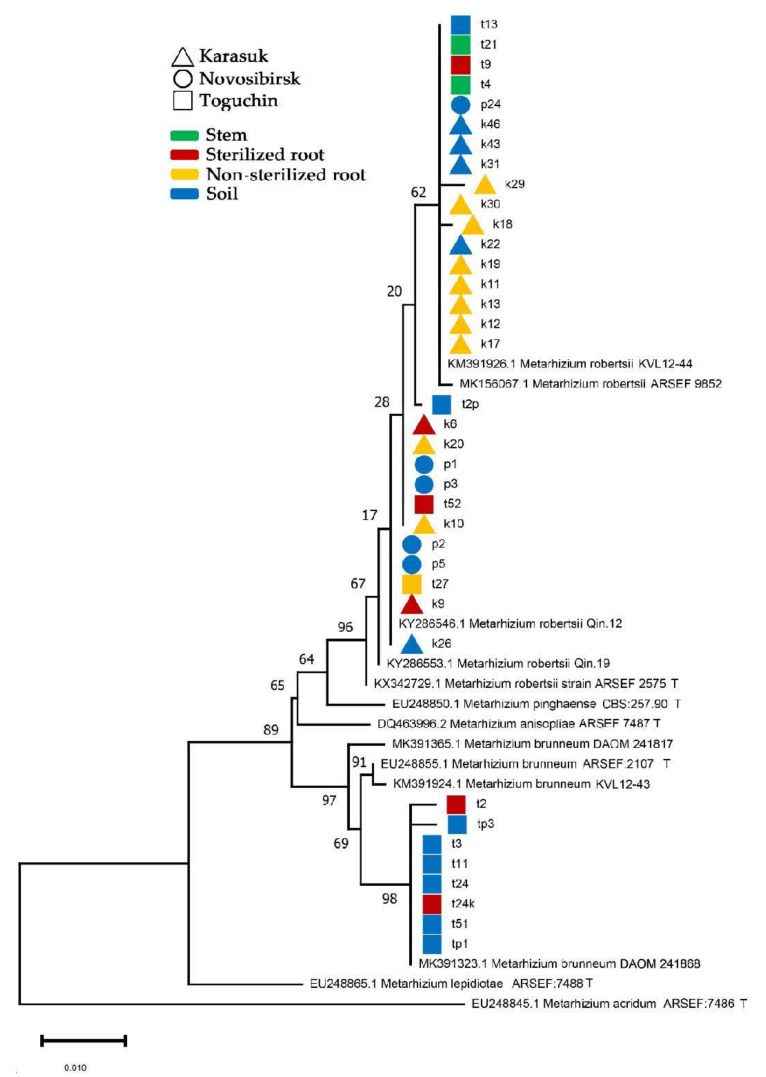
Phylogenetic reconstruction showing the position of *Metarhizium* isolates based on the *5′EF-1α* region sequence obtained using maximum likelihood based on the Tamura–Nei model with 1000 replicates; bootstrap support is indicated on the branches.

Thirty-seven isolates of *Metarhizium* spp. (8 from sterilized plant tissues, 11 from the non-sterilized roots, and 18 from soils) were identified (Figure 4). Two species, *M. robertsii* and *M. brunneum*, were detected with a 29:8 ratio. *M. robertsii* was found in the soil, sterilized and non-sterilized roots, and potato stems, but *M. brunneum* was isolated only from soil and sterilized roots. It should be noted that *M. robertsii* was found in all the locations, while *M. brunneum* was detected only in Toguchin (Fisher’s exact test, *p* = 0.03), i.e., only in silty clay soils and plants from the northeastern location. No significant differences were revealed between the frequencies of *M. robertsii* and *M. brunneum* in the sterilized and non-sterilized roots and stems (Fisher’s exact test, *p* > 0.12).

## 4. Discussion

The present work has shown that endophytic colonization of potato plants was observed only rarely under natural loads of *Beauveria* and *Metarhizium* in the soils. The fungal communities in the internal tissues of potato roots and leaves consisted primarily of phytopathogenic fungi, while the DNA of entomopathogenic fungi was found in negligible amounts. This result indicated that endophytic colonization of potato by entomopathogens is not a stable phenomenon in agrosystems with a natural abundance of entomopathogenic fungi.

The CFU count of *Beauveria* and *Metarhizium* in the studied potato field was 10^2^–10^4^ per gram of dry soil, which was consistent with studies in other agrosystems [15], as well as in potato plantations [46]. The abundance of *Metarhizium* was higher than that of *Beauveria* in the investigated fields. It was shown earlier that *Metarhizium* species were more often isolated from agricultural soils, while *Beauveria* species were isolated either from both agricultural and natural ecosystems [47,48] or primarily from natural and semi-natural habitats [49].

The domestic potato agrosystems (the kitchen gardens in Karasuk and Toguchin) were characterized by the highest CFU counts of entomopathogenic fungi in soil compared with conventional agrosystems (farms in Novosibirsk), which is consistent with other studies on fungal abundance in organic and conventional fields [19,21,50]. Notably, the Novosibirsk and Karasuk locations had similar granulometric and chemical characteristics for their soils, but a significantly higher CFU count was registered in the Karasuk soil. The lowest abundance of the fungi found in the conventional Novosibirsk fields may have been due to intensive tillage, the low density and diversity of weeds, and the low abundance of pest insects. The tillage and reduced weed cover area led to an increase in UV radiation and temperature fluctuations at the surface of the soil, as well as a decrease in the plant root density in the soil, which may have had a negative effect on the fungi. The CFU counts in the soils of both Toguchin and Karasuk were close, although the first location was characterized by silty clay soil, which was also richer in nitrogen and macroelements. Silty and clay soils are usually more favorable for the persistence of entomopathogenic fungi compared with sandy soil, as reviewed by Jaronski [16]. However, a prolonged outbreak of the Colorado potato beetle in the Karasuk location might have led to a higher abundance of entomopathogenic fungi.

There are many examples of successful colonization of Solanaceae plants with entomopathogenic fungi in laboratory assays under cultivation on sterile substrates [51,52,53,54], including potato colonization after foliar spraying [11,55] or introducing fungi into the substrate [12]. In the present field assay, we observed few entomopathogenic fungi isolation events from the internal tissues of potato plants (≈3% *Metarhizium*- and *Beauveria*-positive plants). However, the frequency of *Metarhizium* isolation might reach 53% when nonsterile roots were plated on media. Probably, *Metarhizium* colonizes the potato rhizosphere and rhizoplane, but penetration into internal tissues is rarely observed, especially under field conditions. Moonjely and Bidochka [56] showed that colonization of the rhizoplane with *Metarhizium* species was observed in tomatoes and peppers when grown on vermiculite, but endophytic colonization was detected only in peppers. A similar phenomenon was observed when fungi colonized other plants. Barelli and coworkers [57] grew the common bean *Phaseolus vulgaris* in the laboratory in field-collected soil. An analysis of the dataset for this study showed that the relative abundance of *Metarhizium* OTUs in non-sterilized roots was 0.38–0.51%, and, in the rhizosphere soil, it was 0.85–1.3%. Interestingly, the additional application of *M. robertsii* increased its OTU abundance in the rhizosphere soil but not in the roots. The low level of plant colonization with *Metarhizium* and *Beauveria* was registered by researchers using cultivable methods. For example, Stuart and coworkers [58] showed the absence of *Metarhizium* and *Beauveria* among the dominant endophytes of soybean (*Glycine max*) leaves. Pimentel et al. [6] registered a low percentage of the entomopathogenic fungi *Paecilomyces* and *Beauveria* (no more than 5% of the total number of endophytes) isolated from the sterilized leaves and stems of *Zea mays* in the field and under greenhouse conditions. A microbiological analysis of young plantings of *Carpinus caroliniana* and *Coffea arabica* showed similar results [59,60].

The low level of plant colonization with entomopathogenic fungi may be explained by competition with other endophytes, including phytopathogens [3]. Based on metagenomic sequencing, we showed the predominance of phytopathogenic fungi (*Fusarium, Cladosporium, Alternaria*, and *Rhizoctonia*) in potato roots and leaves, while the relative abundance of *Beauveria* and *Metarhizium* did not exceed 0.3%. A study on the fungal communities in the rhizosphere and rhizoplane of potatoes was recently conducted by Mardanova and coworkers [61] in Tatarstan (Russia). The authors showed the prevalence of Ascomycota (classes Sordoriomycetes and Dothideomycetes), Basidiomycota (class Agaricomycetes), and Zygomycota (class Mortierellomycetes) in the rhizoplane and rhizosphere of potatoes cultivated on an Alfisol soil. This result is close to that of the community of potato endophytes established in the present work. The predominant genera in the rhizoplane of the potatoes in Tatarstan were *Fusarium, Monographella, Chaetomium*, and *Mortierella*. However, the authors also registered a relatively high abundance of *Metacordyceps* (up to 5%). An analysis of the nucleotide archive from this study [62] showed that the OTUs of *Metacordyceps* that were found in the rhizosphere and rhizoplane belonged to *M. chlamydosporia*, a nematode pathogen capable of colonizing plant roots [56]. The abundance of a single OTU belonging to the *Metarhizium anisopliae* complex was only 0.15–0.37% in Tatarstan [62]. OTUs from the *Beauveria* species were not detected. Unfortunately, the internal root tissues were not analyzed by the authors.

We observed significant differences in the relative abundance of phytopathogenic fungi in potatoes from different locations, which can be explained by climatic and soil conditions, as well as agricultural practices. In particular, the high abundance of *Fusarium* in roots and *Alternaria* in leaves from the Karasuk location (steppe zone) is probably associated with the most arid climate. It is known that infection of potatoes with *Fusarium* and the species diversity of these fungi are higher in warm climates [63]. Regarding *Alternaria*, water stress in plants, high wind speed, and a high density of phytophages are characteristics of the Karasuk location. These factors contribute to the dissemination of *Alternaria* conidia and incidence of Solanaceae plants [64]. The predominance of unclassified Pyronemataceae and the decrease in the abundance of *Fusarium* and *Cladosporium* in potato roots from the Novosibirsk farm can probably be explained by the crop rotation. The long-term cultivation of certain plants without rotation leads to the accumulation of phytopathogens, while rotation partially decreases this accumulation [64,65]. Therefore, we see the predominance of phytopathogens in the domestic agrosystems of Karasuk and Toguchin and their lower abundance in the conventional agrosystem of the Novosibirsk location.

Through the sequencing of the *5’EF-1α* region, we showed the presence of two cryptic species, *M. robertsii* and *M. brunneum,* in the potato agrosystems of the studied region. *M. robertsii* was isolated from the soils and plants (roots and stems) in all locations, while *M. brunneum* was isolated from soil and potato roots in the Toguchin location only, i.e., the northeastern point, which is characterized by silty clay soil and high humidity. Previous studies [26,66] indicated that *M. robertsii* is adapted to a wide range of temperatures (including 35–37 °C) and low humidity, while *M. brunneum* is a more mesophilic species that cannot grow at high temperatures and demonstrates low virulence under arid conditions. Therefore, *M. brunneum* was not detected in the arid fields of the steppe zone. Most likely, *M. brunneum* persists better in silty clay soils with high moisture retention.

It is important to note that the low level of potato colonization by entomopathogenic fungi under their natural load does not exclude the benefits for plants following the artificial introduction of the fungi into agrosystems. We showed previously [14] that the pre-planting treatment of tubers with *M. robertsii* and *B. bassiana* suspensions (5 × 10^7^ conidia/mL) led to successful endophytic colonization of potato roots under field conditions (32–47% fungi-positive plants in the flowering period). Potato growth stimulation, immunity activation, and a decrease in *Rhizoctonia* diseases were also registered in treated plants. Most likely, high concentrations of entomopathogenic fungi at the initial stage of potato growth played a key role in the subsequent colonization and successful competition with phytopathogens. Some authors registered a decrease in the level of colonization with entomopathogenic fungi during plant development; in particular, at several months after inoculation, entomopathogenic fungi may not be detected in plants [24,67]. Future studies should focus on the dynamics of potato colonization by entomopathogenic fungi and the changes in fungal communities during the plant development process.

In conclusion, this was the first study to provide a quantitative estimation of entomopathogenic fungi in potato agrosystems. Soils from the conventional fields were characterized by a lower abundance of *Metarhizium* and *Beauveria* compared with soils from kitchen gardens. Three species were isolated from the internal tissues of potato plants, namely, *M. robertsii*, *M. brunneum*, and *Beauveria* sp. Based on our results, there is no reason to consider the entomopathogenic fungi *Metarhizium* and *Beauveria* to be stable endophytes of potato plants in agrosystems with a natural abundance of these fungi in soil, at least in Western Siberia. Fungal isolation from internal potato tissues was sporadic, and the relative fungal abundance was low. The fungal communities of potato roots and leaves were represented primarily by phytopathogens, and their structure depended on the climatic conditions as well as on agricultural practices. However, we should not negate the beneficial effects of entomopathogenic fungi on potatoes following their introduction into agrosystems. Further research could focus on studying the microorganism communities in potato plants after treatment with *Metarhizium* and *Beauveria* fungi.

## Figures and Tables

**Table 1:** Endophytic colonization of potato plants with entomopathogenic fungi in different locations of Western Siberia: Karasuk (K-k), Novosibirsk (N-sk), and Toguchin (T-n).

	% of Fungus-Positive Plants (*n* = 30 per Location)								
Genus	Root			Stem			Leaf		
	K-k	N-sk	T-n	K-k	N-sk	T-n	K-k	N-sk	T-n
2019									
*Metarhizium*	6.7	3.3	3.3	0	0	3.3	0	0	0
*Beauveria*	6.7	0	0	3.3	0	0	0	0	0
2020									
*Metarhizium*	3.3	0	3.3	0	0	10.0	0	0	0
*Beauveria*	0	3.3	0	3.3	0	3.3	0	3.3	0

## Data Availability

The MiSeq data were deposited in GenBank under the study accession number PRJNA729459. Metagenomic data are also presented in Supplementary File S1. Other data are available upon request to the authors.

## Supplementary Materials

The following are available online at https://www.mdpi.com/article/10.3390/microorganisms9071373/s1, Figure S1. title, Location of plots in the Novosibirsk region. Figure S2. Average long-term temperatures and precipitation at the surveyed locations. Figure S3. Content of carbon (Cmic) and nitrogen (Nmic) in the microbial biomass from the investigated soils as analyzed by the fumigation–extraction method. Figure S4. Rarefaction analysis of the investigated samples. Table S1. Granulometric composition of soil samples from experimental plots, as determined by laser diffraction using a Fritsch Analysette-22 MicroTec device. Table S2. Agrochemical properties of soils from the experimental plot locations. File S1. MiSeq data at the OTU, genus, order, class, and phylum levels.
