# Supplementary material for: Can Potato Plants Be Colonized with the Fungi Metarhizium and Beauveria under Their Natural Load in Agrosystems?
Source: Microorganisms. 2021 Jun 24;9(7):1373. doi: 10.3390/microorganisms9071373 (PMC8306205; doi:10.3390/microorganisms9071373)
Supplement: Supplementary file 1 [file microorganisms-09-01373-s001.zip › microorganisms-1255735-suppl/ESM Fig.1-4, Tab 1-2.pdf]

ELECTRONIC SUPPLEMENTARY MATERIAL

Can potato plants be colonized with the fungi *Metarhizium* and *Beauveria* under their natural load in agrosystems?

Maksim Tyurin[1]*, Marsel R. Kabilov[2], Natalia Smirnova[3], Oksana G. Tomilova[1], Olga Yaroslavtseva[1], Tatyana Alikina[2], Viktor V Glupov[1], Vadim Yu Kryukov[1]*

1 Institute of Systematics and Ecology of Animals, Siberian Branch of the Russian Academy of Sciences, Novosibirsk, 630091, Russia
2 Institute of Chemical Biology and Fundamental Medicine, Siberian Branch of Russian Academy of Sciences, Novosibirsk, 630090, Russia
3 Institute of Soil Science and Agrochemistry, Siberian Branch of the Academy of Sciences, Novosibirsk, 630090, Russia
* Correspondence: V. Yu. Kryukov krukoff@mail.ru; M. Tyurin, maktolt@gmail.com

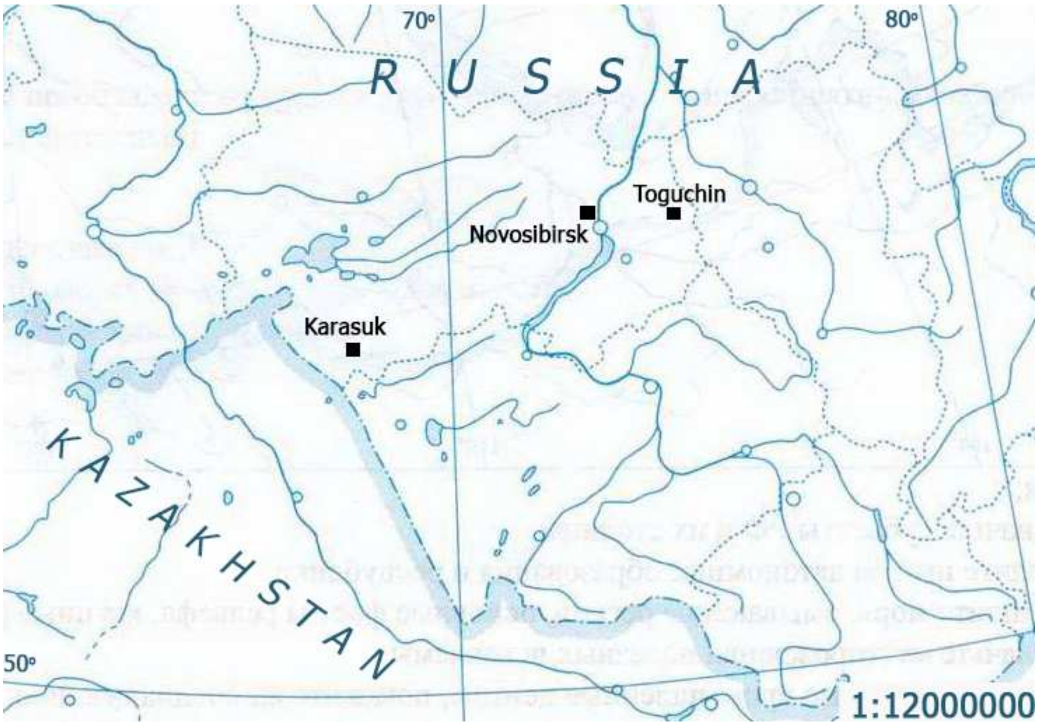

Figure S1. Location of plots in the Novosibirsk region.

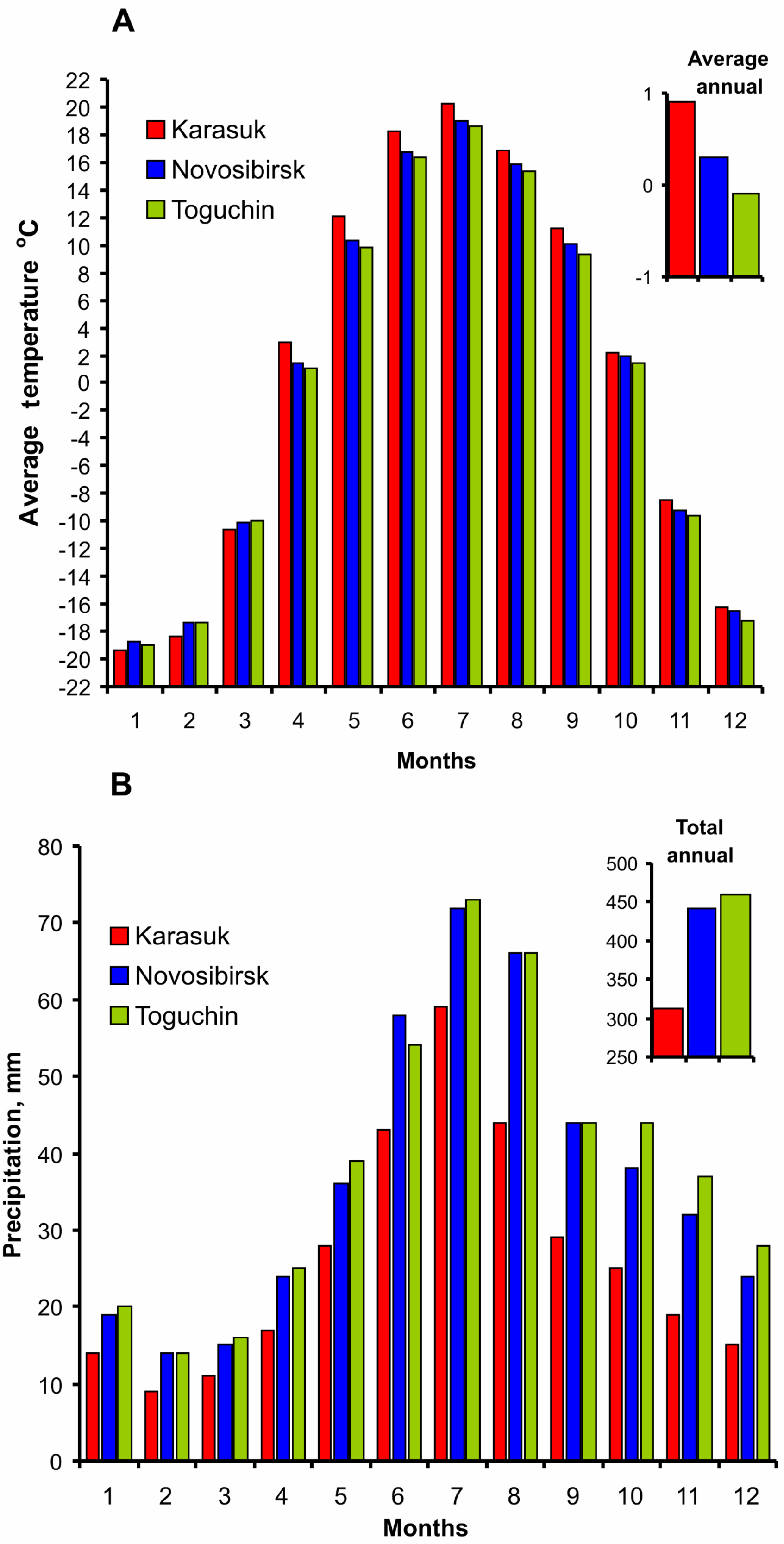

Figure S2. Average long-term temperatures and precipitation in the surveyed locations. Plots generated based on data from Kravtsov and Donukalova [1].

Table S1. Granulometric composition of soil samples from experimental plots, as determined by laser diffraction using a Fritsch Analysette-22 MicroTec device.

Location of plots	Sludge weight - particles diameter more than 250 µm, g	Percent of particles with different diameter						Physical clay < 10 µm	Physical sand > 10 µm
		1000-250 µm	250-50 µm	50-10 µm	10-5 µm	5-1 µm	1-0,1 µm		
Karasuk	2.1±0.1	15.8±1.0	11.7±1.6	24.7±2.4	15.0±4.7	25.3±2.4	7.6±0.9	47.8±4.7	52.2±4.7
Novosibirsk	0.06±0.01	0.6±0.1	27.1±1.9	39. 8±1.4	11.1±2.8	16.8±1.4	4.6±0.5	32.6±2.8	67.4±2.8
Toguchin	0.06±0.03	0.4±0.2	2.0±0.2	36.4±5.5	22.6±9.2	33.6±5.5	5.7±0.7	61.9±9.2	38.1±9.2

Table S2. Agrochemical properties of soils in the location of experimental plots.

Soil properties*	Content in 5-20 cm layer		
	Karasuk	Toguchin	Novosibirsk
$pH_{(H2O)}$	5.2 ± 0.5	6.7 ± 0.5	7.5 ± 0.1
C_{org}, %	1.35 ± 0.3	5.2 ± 0.2	0.8 ± 0.04
N_{tot}, %	0.15 ± 0.04	0.5 ± 0.03	0.09 ± 0.01
$N\text{-}NO_3$, mg kg^{-1}	18.2 ± 5.5	34.3 ± 7.6	17.3 ± 8.1
$N\text{-}NH_4$, mg kg^{-1}	0.94 ± 0.5	1.7 ± 1.3	0.7 ± 0.2
$Phosphorus_{mobile}$, mg P_2O_5 g^{-1}	14.9 ± 3.9	6.7 ± 0.5	7.5 ± 0.1
Content of mobile forms of macroelements in acetate-ammonium buffer			
Calcium, (mg Ca/100 g^{-1})	130.5 ± 6.7	443.9 ± 23.9	297 ± 8.9
Magnesium, (mg Mg/100 g^{-1})	27.1 ± 1.3	67.1 ± 5.7	16.1 ± 1.3
Potassium, (mg K/100 g^{-1})	93.1 ± 22.0	227.3 ± 15.6	12.4 ± 2.1
Sodium, (mg Na/100 g^{-1})	3.4 ± 0.9	4.8 ± 0.3	2.5 ± 0.3

* - pH of the aqueous soil suspension (1 : 2.5) was analyzed by potentiometry. The carbon (Corg) content was analyzed after wet burning in a mixture of 0.4 N $K_2Cr_2O_7$ with H_2SO_4[2]. The total nitrogen content (Ntot) was determined by the Kjeldahl technique after wet ignition of the samples with the Kudeyarov catalyzing reducing agent [3]. Exchangeable ammonium ($N\text{-}NH_4$) was detected by Kudeyarov method after extraction with 0.1 N KCl [4]. Nitrate nitrogen ($N–NO_3$) and phosphorus (P_2O_5), were detected on the basis of extraction with 0.003 M K_2SO_4. Exchangeable K^+, Na^+, Ca^{2+} and Mg^{2+} were determined by atomic adsorption method after extraction with 1M CH_3COONH_4 [5-6]. All analyses were performed in three replicates.

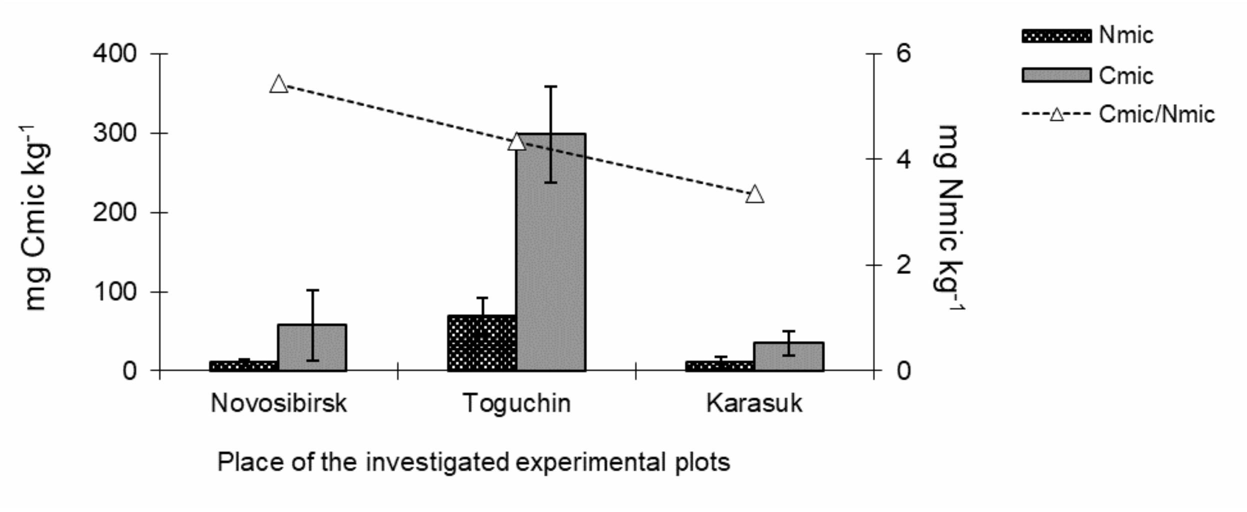

Figure S3. Content of carbon (Cmic) and nitrogen (Nmic) of the microbial biomass in the investigated soils as analyzed by fumigation-extraction method [7-8]. Analyses were performed in three replicates.

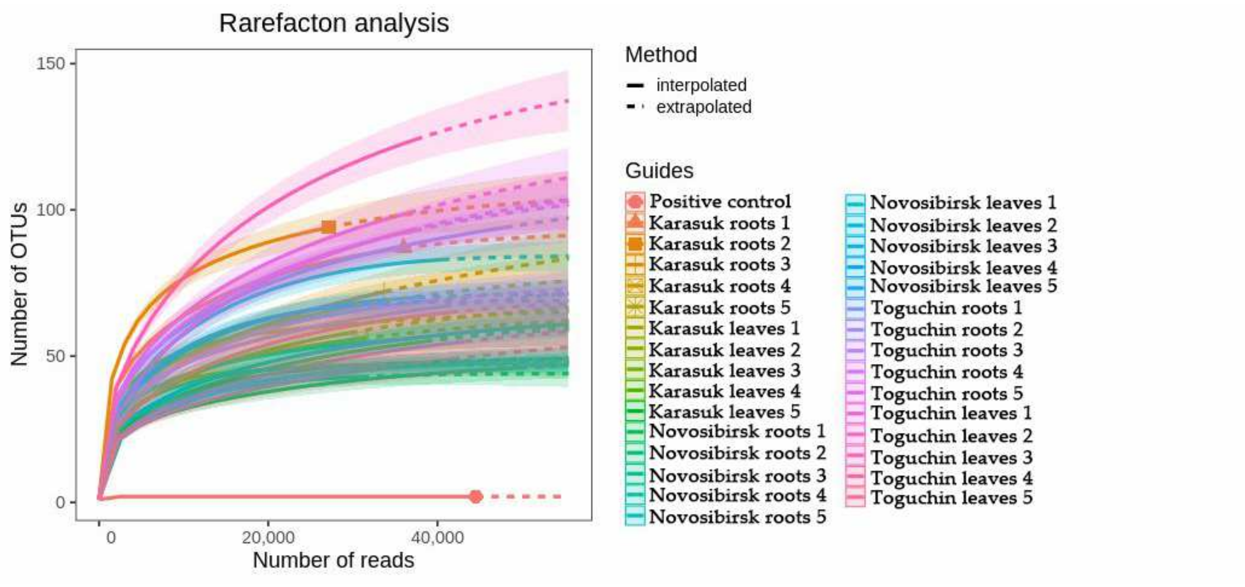

Figure S4. Rarefaction analysis of the investigated samples. Plastid and mitochondrial reads were not excluded.

References

1. Kravtsov, V.M.; Donukalova, R.P. Geography of Novosibirsk region. INFOLIO, Novosibirsk. **1999**, 208 pp. (in Russian).
2. Sokolov, A.V. Agrochemical Methods of Soil Studies. Nauka, Moscow. 1975; pp. 656. (in Russian).
3. Ma, T.S.; Zuazaga, G. Micro - Kjeldahl determination of nitrogen. A new indicator and an improved rapid method. *Ind. Eng. Chem. Anal. Ed*., 1942, *14*, 280–282.
4. Maslova, A.L. Potassium as an element of soil fertility, in: Potassium fertilizers. LOVIUAA, Leningrad, 1938. pp. 82-171 (in Russian).
5. Kudeyarov, V.N. Colorimetric determination of ammonium nitrogen in soils and plants phenolic methods. *Agrochemistry*, 1965, *6*, 146-150 (in Russian).
6. Blackemore, L.C.; Searle, P.L.; Daly, B.K. Methods for chemical analysis of soils. *New Zealand Soil Bureau scientific report*; 10A. 1981. 102 pp. doi: 10.7931/DL1-SBSR-10A
7. Brookes, P.C.; Landman, A.; Pruden, G.; Jenkinson, D.S.; Chloroform fumigation and the release of soil nitrogen a rapid direct extraction method to measure microbial biomass nitrogen in the soil. *Soil Boiol. Biochem.*, 1985. *17*. 837-842.
8. Vance, E.D.; Brookes, P.C.; Jenkinson, D.S.; An extraction method for measuring soil microbial biomass-C. *Soil Biol. Biochem*., 1987. *19*. 703-707.
